# Successful management of splenic artery dissection after sigmoid colon perforation in vascular Ehlers–Danlos syndrome

**DOI:** 10.1186/s40792-024-01845-6

**Published:** 2024-03-15

**Authors:** Moegi Yoshizaki, Yasuko Matsuo, Satoshi Yasuda, Shunsuke Doi, Takeshi Sakata, Minako Nagai, Kota Nakamura, Yuichiro Kohara, Shohei Toyoda, Toshihiro Tanaka, Masayuki Sho

**Affiliations:** 1 Department of Surgery, Nara Medical University, 840 Shijo-Cho, Kashihara, Nara 634-8522 Japan; 2 Department of Radiology, Nara Medical University, 840 Shijo-Cho, Kashihara, Nara 634-8522 Japan

**Keywords:** Vascular Ehlers–Danlos syndrome, Intestinal perforation, Arterial rupture

## Abstract

**Background:**

Ehlers–Danlos syndrome (EDS) is a genetic disorder that causes fragility of the systemic connective tissues. Of the 13 subtypes, vascular EDS (vEDS) is associated with abnormalities in collagen production, resulting in arterial rupture and intestinal perforation. Herein, we report the case of a man with confirmed vEDS who survived a ruptured dissected splenic artery aneurysm triggered by perforation of the sigmoid colon.

**Case presentation:**

A 48-year-old man presented to our hospital with sudden severe lower abdominal pain. The patient was genetically diagnosed with vEDS at the age of 43 years. Abdominal computed tomography (CT) showed fluid and free air surrounding the sigmoid colon. These findings suggested sigmoid colon perforation, and emergency surgery was needed. Hartmann’s procedure was performed. The resected specimen showed a 2-cm-sized depression around the perforation. Histopathological findings showed an abscess and exudate in the serosa of the perforation and thinning of the intrinsic muscular layer in the depressed area. The patient was doing well postoperatively; however, on the ninth postoperative day, sudden upper abdominal pain developed. CT revealed an intra-abdominal hemorrhage due to rupture of a dissecting splenic artery aneurysm. The aneurysm was not observed on preoperative CT and was distant from the surgical site. Urgent transcatheter arterial embolization was performed. Although embolization of the splenic artery was attempted during the procedure, the arterial dissection spread to the common hepatic artery. Moreover, the proper hepatic and gastroduodenal arteries were poorly visualized, probably due to vasospasm. Although complications associated with extensive embolization were a concern, embolization of the splenic and common hepatic arteries was necessary to save the patient’s life. After embolization, angiography showed that the left hepatic blood flow was maintained from the inferior phrenic artery, and the right hepatic inflow was maintained from the superior mesenteric artery via the peribiliary vascular plexus in the hilar area. The patient recovered well and was discharged on the 19th postoperative day.

**Conclusions:**

vEDS can cause arterial rupture after intestinal surgery. Therefore, careful post-operative management is necessary. Moreover, cooperation with interventional radiologists is important for prompt treatment of vascular complications.

## Background

Ehlers–Danlos syndrome (EDS) is a genetic disorder that causes systemic connective tissue fragility in the skin, joints, and blood vessels [1] with a frequency of approximately 1/50,000–1/200,000 based on the EDS subtypes [2]. This is caused by mutations in genes encoding specific collagen molecules or enzymes required for collagen maturation. In 2017, the International EDS Consortium published a new international classification system that recognized 13 subtypes to replace the Villefranche classification [3]. Of these, vascular EDS (vEDS) has an autosomal dominant inheritance pattern and is associated with mutations in the *COL3A1* and/or *COL1A1* genes, which encode type III and type I collagens, respectively. Major clinical criteria include arterial rupture at a young age, spontaneous perforation of the sigmoid colon without diverticular disease or other bowel pathologies, uterine rupture (specifically in the third trimester with no risk factors), formation of a carotid–cavernous sinus fistula without trauma, and a family history confirmed via genetic testing [2]. A previous report showed that the median survival of patients with the vascular subtype was for 48 years. Most deaths were caused by arterial rupture. Bowel rupture accounts for approximately a quarter of complications but rarely leads to death [2]. Herein, we report the case of a man with confirmed vEDS who survived a ruptured dissected splenic artery aneurysm triggered by perforation of the sigmoid colon.

## Case presentation

A 48-year-old man presented to our hospital with the chief complaint of sudden lower abdominal pain. He had a history of right forearm artery rupture and was diagnosed with vEDS by genetic testing at the age of 43 years, which revealed missense variants in *COL3A1*. The patient was followed up regularly at our hospital.

**Fig. 1:**
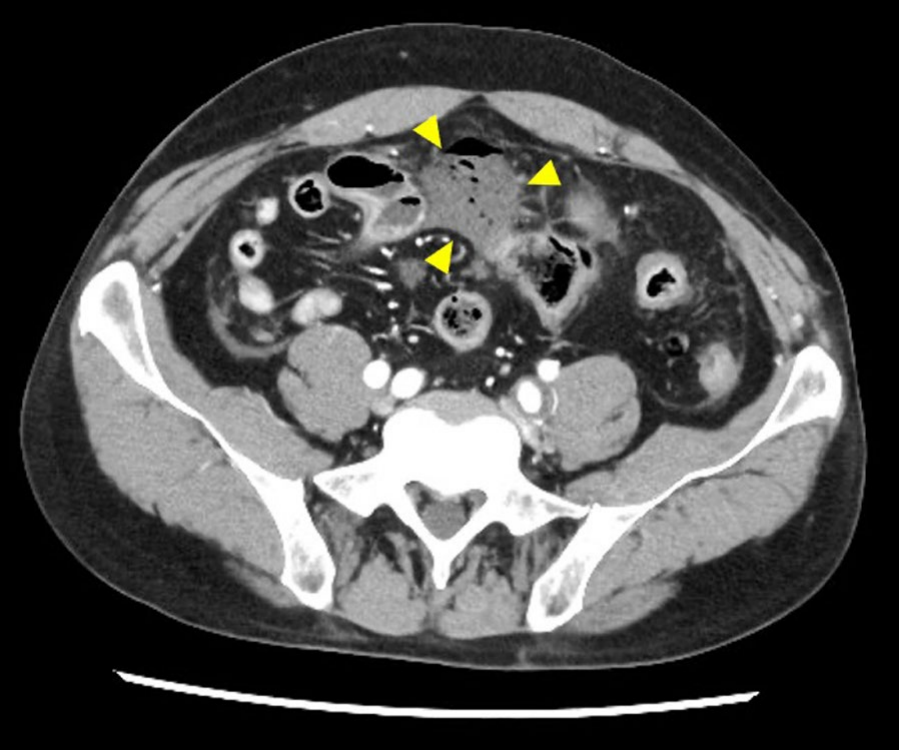
Preoperative abdominal CT findings. Fluid accumulation and free air around the sigmoid colon are observed

**Fig. 2:**
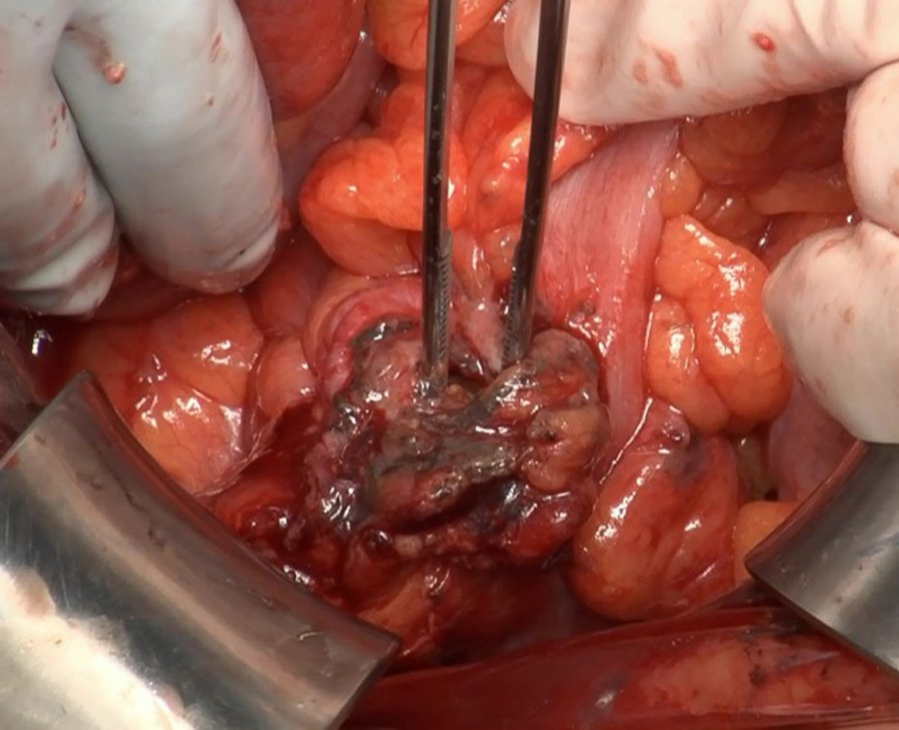
Intraoperative findings. A part of the wall of the sigmoid colon is thinned, and a 7-mm perforation is observed

**Fig. 3:**
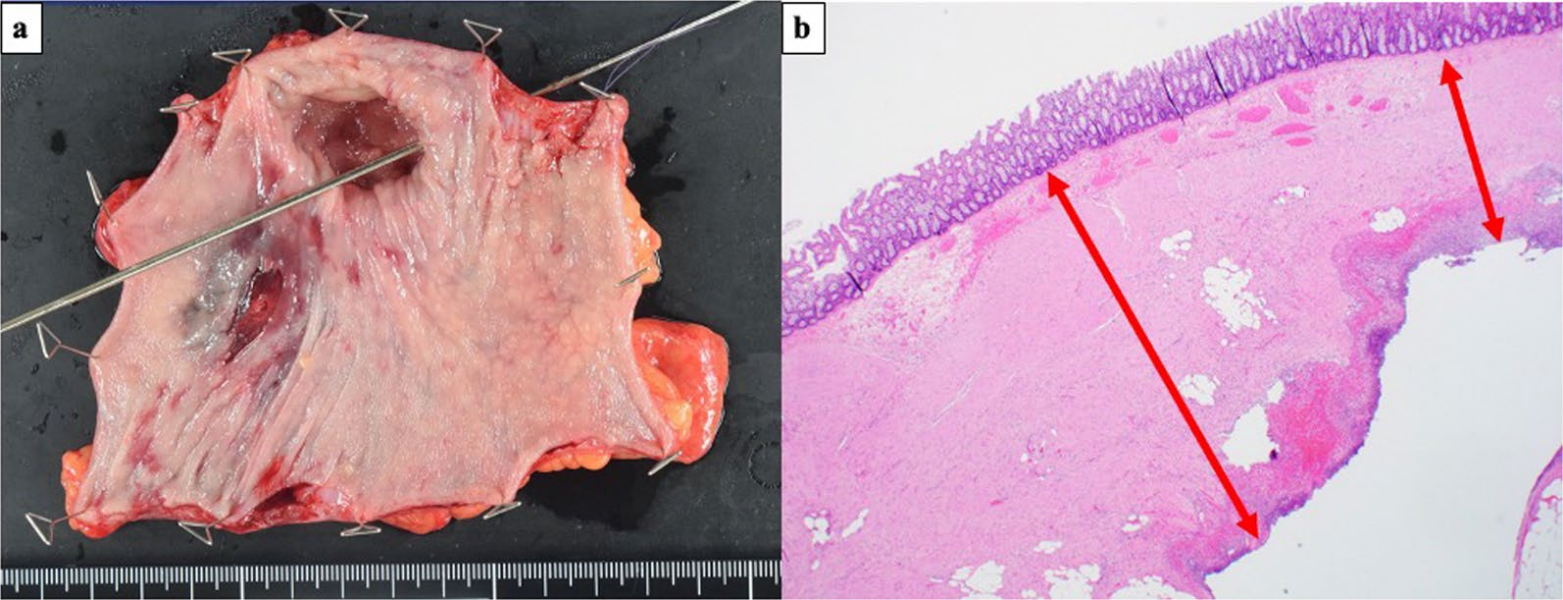
Gross and pathological findings of the specimen. **a** There is 2 cm of wall thinning, with a 7-mm perforated area within it. **b** The intrinsic muscle layer is thinner around the perforation site

Contrast-enhanced computed tomography (CT) of the abdomen revealed ascites and free air, particularly around the sigmoid colon. These findings suggested a sigmoid colon perforation (Fig. 1). Although surgery for a patient with EDS carries the risk of fatal complications, emergency surgery was performed on the same day because the patient developed peritonitis. Contaminated ascetic fluid was observed in the abdominal cavity when the abdomen was opened through a lower abdominal midline incision. Part of the wall of the sigmoid colon was thinned, and a perforation was observed in this area (Fig. 2). The perforated area was covered by the greater omentum or mesenteric fat. We resected a part of the sigmoid colon and constructed a colostomy using the remnant sigmoid colon. We checked the remaining colon by inspection and palpation, and found several areas of wall weakening. Because these areas were considered to be at risk of future perforations, we reinforced them with seromuscular layer sutures. A drain was placed after washing the abdominal cavity with 10 L of saline. The operative time was 208 min, and the intraoperative blood loss was 830 ml. The tissue was very fragile and even slight traction could cause tissue damage and hemorrhage; therefore, surgery was performed cautiously. The specimen after sigmoid colon resection showed a 2-cm-sized depression around the perforation (Fig. 3a). Histopathological findings showed an abscess and exudate in the serosa of the perforation and thinning of the intrinsic muscular layer in the depressed area (Fig. 3b).

**Fig. 4:**
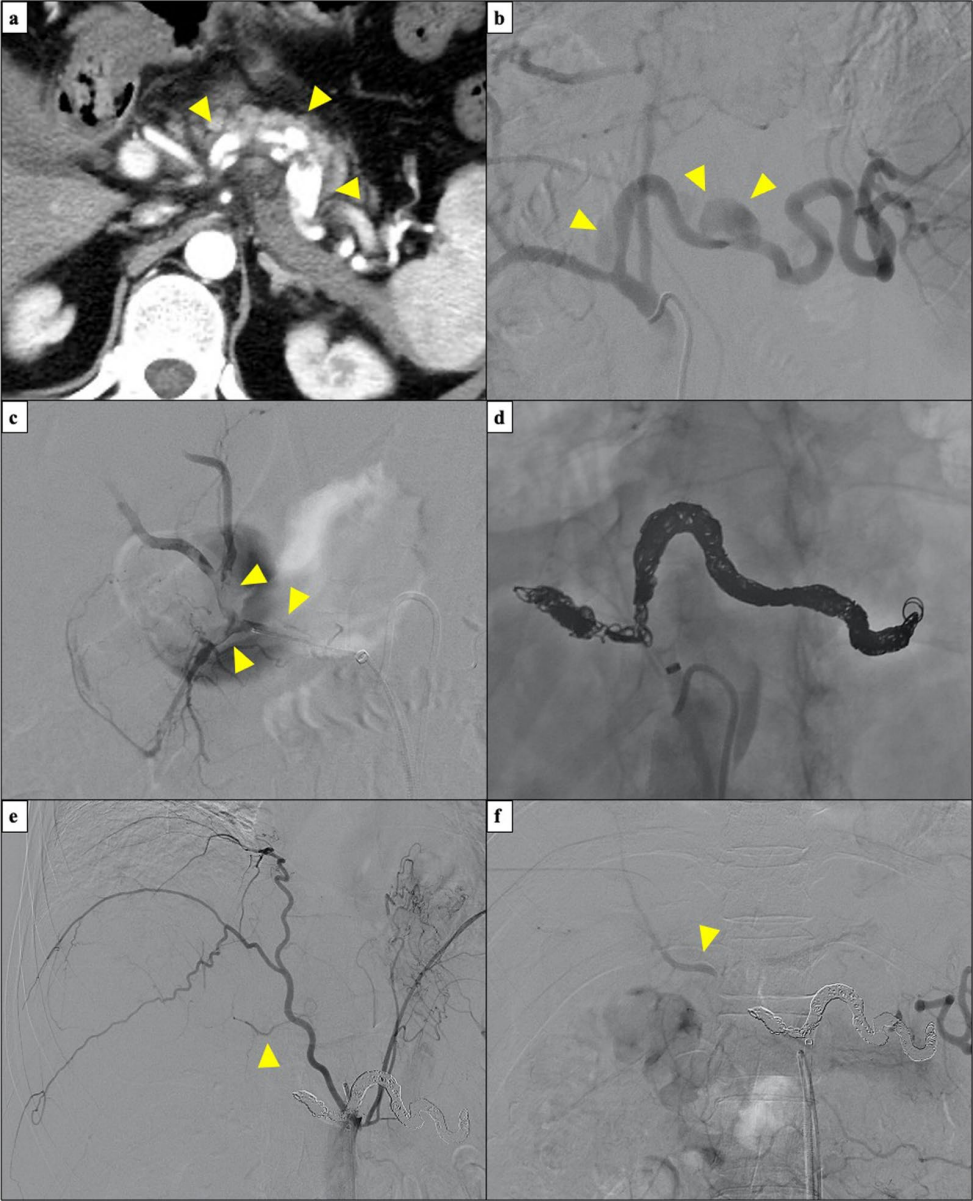
Findings at the time of splenic artery dissection. **a**, **b** Enhanced CT shows intra-abdominal hemorrhage due to rupture of a dissecting splenic artery aneurysm. **c** Arterial dissection spread to the common hepatic artery. **d** Splenic and common hepatic arteries are embolized. **e** Left hepatic blood flow is maintained from the inferior phrenic artery. **f** Right hepatic inflow is maintained from the superior mesenteric artery via peribiliary vascular plexus in the hilar area

The patient was doing well postoperatively. However, on the ninth postoperative day, the patient developed sudden abdominal pain with cold sweats and decreased blood pressure. Enhanced CT revealed intra-abdominal hemorrhage due to the rupture of a dissecting aneurysm in the splenic artery (Fig. 4a), which was distant from the surgical site. Preoperative CT showed no splenic artery aneurysm. Rupture was not considered to be caused by direct surgical invasion. After an immediate consultation with an interventional radiologist, urgent transcatheter arterial embolization (TAE) was performed. First, the splenic artery was embolized (Fig. 4b). However, during catheterization, the arterial dissection spread to the common hepatic artery, root of the proper hepatic artery, and gastroduodenal artery, owing to the fragility of the arterial wall (Fig. 4c). Although the complications associated with extensive embolization were a concern, embolization of these arteries was necessary to save the patient’s life (Fig. 4d). After embolization, angiography showed that the left hepatic blood flow was maintained from the inferior phrenic artery (Fig. 4e), and the right hepatic inflow was maintained from the superior mesenteric artery via the peribiliary vascular plexus in the hilar area (Fig. 4f). After TAE, the patient had no liver dysfunction or ischemia of the gastrointestinal tract, and was discharged from the hospital on the 19th postoperative day.

## Discussion

Spontaneous perforation of the sigmoid colon and arterial rupture are clinical symptoms included in the diagnostic criteria for vEDS. The most notable feature of this case was the arterial rupture observed in the early postoperative period. Similar to the present case, a case of arterial rupture at a site not directly related to surgery after surgical treatment of a sigmoid perforation has been reported; however, the patient died [4]. In our case, rupture was diagnosed early using contrast-enhanced CT, and TAE was performed immediately, which saved the patient’s life.

Idiopathic perforation of the sigmoid colon is the most common intestinal complication associated with vEDS. Although it is an indication for emergency surgery, it is important to recognize that the perioperative complications and mortality rates are very high because of tissue fragility and poor wound healing [2]. Moreover, repeat perforation rates of up to 55% have been reported after segmental resection [2]. To date, appropriate surgical procedures have not been clearly defined in the literature. Speak et al. suggested in their systematic review that the safest approach for intestinal perforation in patients with EDS is total abdominal colectomy to prevent reperforation. However, they also reported that there is no currently available evidence to suggest that such a strategy is likely to be beneficial, and that it seems most likely to be applicable to patients with an abnormally dilated colon [5]. In this case, Hartmann operation was performed because of the large perforation site, strong contamination, and risk of anastomotic leakage. The Hartmann procedure is useful in cases with a high risk of suture failure, such as gastrointestinal perforation, abscess, and malnutrition and is the technique of choice, especially in emergency surgery [6]. Instead of preserving the colon, to avoid reperforation of the intestine after surgery, we checked whether there were areas of wall weakening in the remnant colon.

Arterial rupture is the most common complication of vEDS; it is fatal in acute settings and accounts for most deaths. Management of vascular complications in patients with vEDS is challenging. Owing to the fragility of the arterial wall, open surgery and endovascular treatment have high complication rates and mortality risks of 30% and 24%, respectively [7]. In recent years, endovascular treatment has become the treatment of choice for arterial complications. Shalhub et al. summarized the treatment of 88 patients with splenic arterial aneurysms in vEDS and reported that open repair of the ruptured splenic arterial aneurysm was associated with high morbidity and mortality, whereas embolization was associated with favorable outcomes with none of the patients experiencing periprocedural mortality or access site complications [8]. However, they also described several technical considerations that should be considered during endovascular approaches for patients with vEDS. These include gentle intraoperative wire manipulation to avoid iliac or other arterial dissections, using the smallest diameter sheath possible, using microcatheters when applicable to limit the risk of intimal injury, and avoiding excessive manipulation of the wires and catheters [9–11]. In the present case, we perform usual procedure of endovascular treatment. Although the arterial dissection progressed during TAE, we were able to rapidly treat the patient and save his life. As mentioned above, we believe that endovascular treatment is the first choice for arterial complications in patients with vEDS. However, endovascular treatment carries some risks, and we presume that treatment in such patients should be performed at facilities with skilled radiologists.

Although both intestinal perforation and arterial rupture are potentially fatal complications, the patient was treated appropriately and successfully. However, the invasive treatment with vEDS is associated with significant risks. Algahtani et al. reported a case of aneurysm rupture of the left hepatic artery 1 week after endovascular treatment for dissection of the left common iliac artery and splenic artery aneurysm. Interactive embolization and hybrid intervention might have enhanced systemic vascular stress, inflammation, and the occurrence of hepatic artery rupture during hospitalization [12]. Moreover, Horowitz et al. observed splenic artery and cardiac ruptures with unknown causes at 3 and 21 days after endovascular treatment, respectively, and referred these events as “remote vascular catastrophes” [13]. The exact cause of these catastrophes is unknown, but elevated collagenase activity after invasive treatment may be a potential cause [2]. These reports, including the present case, indicate that surgeons must always be aware, even in the postoperative period, that vEDS can damage the blood vessels and gastrointestinal tract, which are the major components of type III collagen, during perioperative management. Although no fundamental treatment for vEDS has been established, β-blockers (celiprolol) are recommended for the prevention of arterial complications [14, 15]. Moreover, although there are no clear values for postoperative blood pressure control owing to the small number of cases, blood pressure should be strictly controlled, especially during the postoperative period. Byers et al. recommended controlling blood pressure in the normal or low-normal range and preventing surges in blood pressure to minimize the likelihood of arterial dissection or rupture [16]. Baderkhan et al. showed that high-pulse pressure (> 50 mmHg) may be associated with vascular events and that lowering pulse pressure may be used as a criterion for successful medication in future studies [17]. In this case, the patient was administered celiprolol and an angiotensin receptor blocker before surgery. His systolic blood pressure was approximately 100 mmHg. After surgery, we continued to administer celiprolol and analgesics, and were careful to avoid elevated blood pressure. Although his systolic blood pressure was < 120 mmHg, the splenic artery ruptured 9 days after surgery. After TAE, his systolic blood pressure was strictly controlled to maintain approximately 100 mmHg, and he had no complications. Although these blood pressure changes are not particularly problematic in normal postoperative management, this case reminds us of the need for careful postoperative attention in these patients. The postoperative management of vEDS requires careful monitoring and control of blood pressure using antihypertensive or analgesic drugs. In addition, postoperative complaints of sudden abdominal pain require close examination considering the possibility of arterial rupture and a prompt response in collaboration with an interventional radiologist, as described above.

## Conclusion

In conclusion, surgery for gastrointestinal tract perforation in vEDS requires appropriate surgical technique selection and perioperative management. Surgeons should cooperate with physicians and interventional radiologists to perform surgery in an environment that allows the prompt diagnosis and treatment of postoperative complications.

## Data Availability

No applicable.

## Abbreviations

vEDS: Vascular Ehlers–Danlos syndrome
CT: Computed tomography
TAE: Transcatheter arterial embolization
